# Scraping away technique under cholangioscopy using an improved forceps biopsy device for Mirizzi syndrome

**DOI:** 10.1055/a-2552-0193

**Published:** 2025-03-21

**Authors:** Takeshi Ogura, Takafumi Kanadani, Nobuhiro Hattori, Kimi Bessho, Hiroki Nishikawa

**Affiliations:** 1 38588 Endoscopy Center, Osaka Medical and Pharmaceutical University Hospital, Osaka, Japan; 2 13010 2nd Department of Internal Medicine, Osaka Medical and Pharmaceutical University Hospital, Osaka, Japan

Mirizzi syndrome is a relatively rare disease that causes inflammation of the common bile duct due to an impacted gallstone in the neck of the gallbladder or cystic duct. It occurs in about 1–2% of patients with cholelithiasis 1 2 . The gold standard treatment for Mirizzi syndrome is surgical resection. An alternative technique is electrohydraulic lithotripsy (EHL) under endoscopic retrograde cholangiopancreatography (ERCP) guidance. However, EHL can be indicated if the stone is visible on cholangioscopy. However, in cases of stones coated by gallbladder mucosa, this technique might not be indicated. Recently, an improved forceps biopsy device for the SpyGlass DS was released (SpyBite MAX; Boston Scientific, Marlborough, Massachusetts, USA), with microteeth on the tip of the forceps that improve the tissue gripping force. Technical tips for EHL using this forceps biopsy device, which is called the “scraping-away technique,” to treat Mirizzi syndrome are described.

A 77-year-old man with complications from advanced lung cancer was admitted to our hospital due to obstructive jaundice. On computed tomography (CT), Mirizzi syndrome was diagnosed. Because he had severe heart failure, an endoscopic approach was attempted. After successful biliary cannulation and contrast medium injection, the stone was seen to be impacted around the cystic duct. Because the guidewire was inserted across the stone, the cholangioscope was inserted. Then, the gallbladder mucosa was observed ( Fig. 1 ), and this obstructed bile juice flow. First, mucosa was scraped away using the SPY-Bite MAX ( Fig. 2 ). After this procedure, the stone could be visualized ( Fig. 3 ). Subsequently, EHL was successfully performed ( Fig. 4 ). Then, the guidewire was successfully inserted into the hepatic hilar region across the stone, and two plastic stents were deployed without any adverse events ( Fig. 5 , Video 1 ). No adverse events were observed during the three months until his death due to advanced lung cancer.

**Fig. 1:**
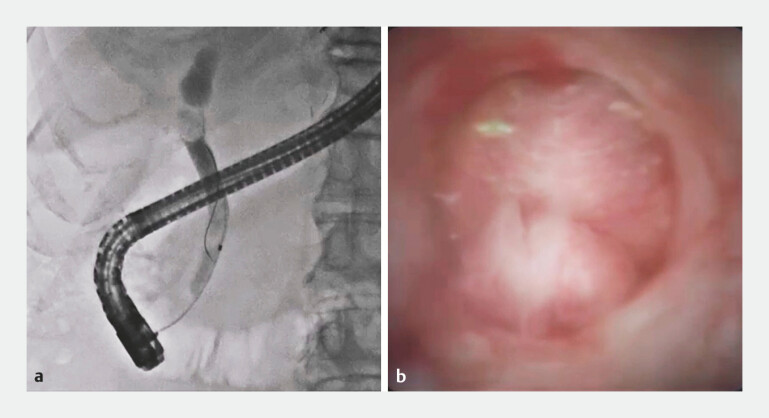
The impacted stone is observed at the confluence between the cystic duct and common bile duct, and the surface of stone may be covered by gallbladder mucosa.

**Fig. 2:**
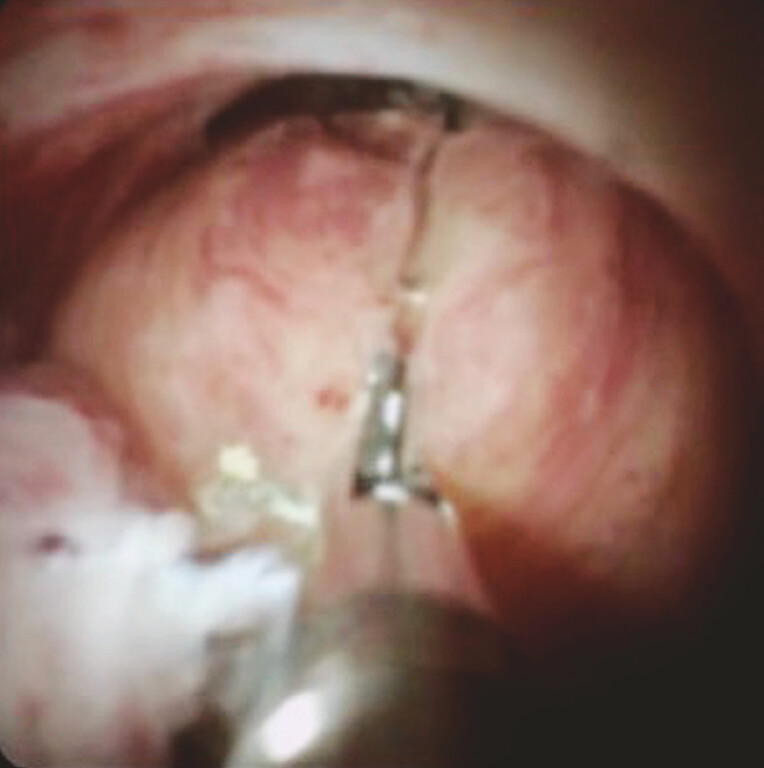
Mucosa is scraped away using the SPY-Bite MAX.

**Fig. 3:**
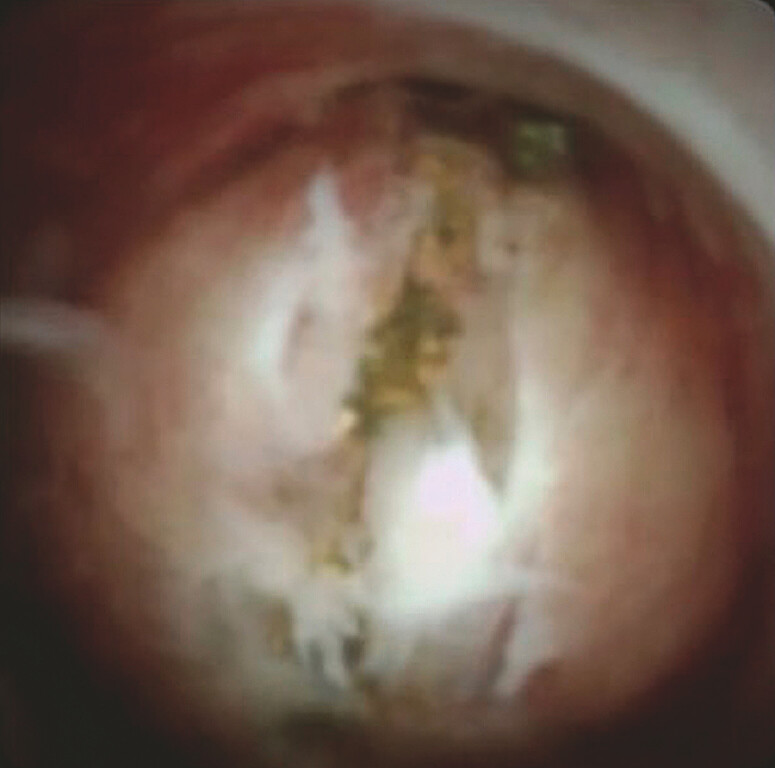
The stone can be visualized.

**Fig. 4:**
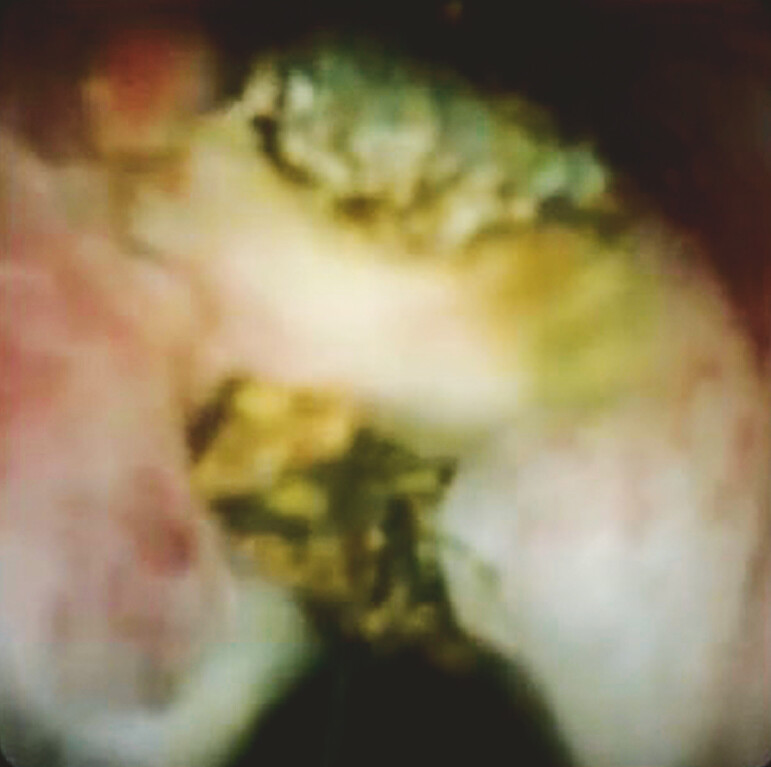
Electrohydraulic lithotripsy is successfully performed.

**Fig. 5:**
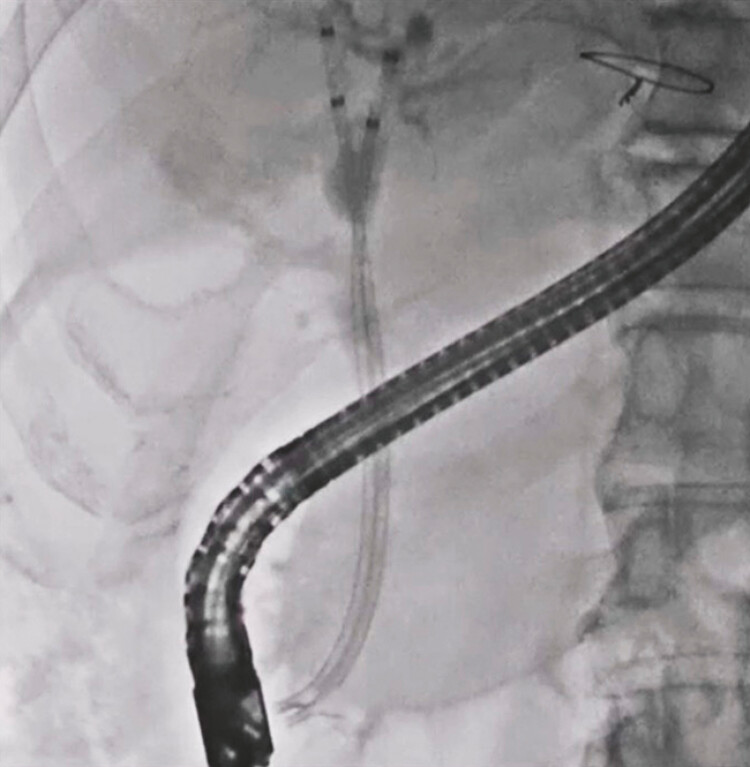
Two plastic stents are deployed without any adverse events.

Scraping-away technique under cholangioscopy using an improved forceps biopsy device for Mirizzi syndrome.Video 1

In conclusion, the scraping-away technique might be useful for treating Mirizzi syndrome if surgical resection cannot be performed.

Endoscopy_UCTN_Code_TTT_1AR_2AH

## Notes

Endoscopy E-Videos https://eref.thieme.de/e-videos
*E-Videos* is an open access online section of the journal *Endoscopy* , reporting on interesting cases and new techniques in gastroenterological endoscopy. All papers include a high-quality video and are published with a Creative Commons CC-BY license. Endoscopy E-Videos qualify for HINARI discounts and waivers and eligibility is automatically checked during the submission process. We grant 100% waivers to articles whose corresponding authors are based in Group A countries and 50% waivers to those who are based in Group B countries as classified by Research4Life (see: https://www.research4life.org/access/eligibility/ ). This section has its own submission website at https://mc.manuscriptcentral.com/e-videos .
